# Role of multimodality cardiac imaging for evaluation of intramyocardial dissection, from dissecting haematoma to false-pseudoaneurysm: a case series

**DOI:** 10.1093/ehjcr/ytae219

**Published:** 2024-05-07

**Authors:** Irene Carrión-Sánchez, Carlos Tejada-González, Jennifer Gómez-Delgado, Rocio Párraga, Javier Cobiella, Jose Alberto de Agustín, Eduardo Pozo-Osinalde, Alberto García-Lledó

**Affiliations:** Cardiology Department, Cardiovascular Institute, Hospital Universitario Clínico San Carlos, Calle del Prof Martín Lagos, S/N, Moncloa - Aravaca, 28040 Madrid, Spain; Cardiology Department, Hospital Universitario Principe de Asturias, Alcalá de Henares, Spain; Cardiology Department, Hospital Universitario Principe de Asturias, Alcalá de Henares, Spain; Cardiology Department, Cardiovascular Institute, Hospital Universitario Clínico San Carlos, Calle del Prof Martín Lagos, S/N, Moncloa - Aravaca, 28040 Madrid, Spain; Cardiac Surgery Department, Cardiovascular Institute, Hospital Universitario Clínico San Carlos, Madrid, Spain; Cardiology Department, Cardiovascular Institute, Hospital Universitario Clínico San Carlos, Calle del Prof Martín Lagos, S/N, Moncloa - Aravaca, 28040 Madrid, Spain; Cardiology Department, Cardiovascular Institute, Hospital Universitario Clínico San Carlos, Calle del Prof Martín Lagos, S/N, Moncloa - Aravaca, 28040 Madrid, Spain; Cardiology Department, Hospital Universitario Principe de Asturias, Alcalá de Henares, Spain

**Keywords:** Intramyocardial dissection, Acute myocardial infarction, Multimodality imaging, Acute cardiac care, Case series

## Abstract

**Background:**

Intramyocardial dissection (ID) is an extremely rare myocardial infarction mechanical complication. Although both clinical and imaging assessment of this rare condition remains a challenge, recent multimodality imaging techniques may help to confirm and to assess the progressive nature of the disease. Diagnosis may be reached in different stages, from as early as the intramyocardial dissecting haematoma to the severe false-pseudoaneurysm.

**Case summary:**

This series describes five cases of ID and provides insights into imaging findings and clinical course of this extremely uncommon condition. Our patients represented a wide range of clinical stages, from asymptomatic course to cardiogenic shock. The imaging diagnostic approach was very different from case to case and involved techniques such as echocardiography, cardiac CT, and cardiac magnetic resonance.

**Discussion:**

Intramyocardial dissection is a challenging condition in terms of diagnosis and clinical management associated with high morbidity and mortality. Furthermore, the different nomenclature found in the literature may be confusing. This case series supports the need of a terminology standardization and a multimodal imaging approach, which might be determinant for an accurate differential diagnosis and a suitable therapeutic management.

**Learning points**

- Intramyocardial dissection is a rare mechanical complication of myocardial infarction that may be presented in different stages.
- Advanced imaging techniques, such as cardiac CT and MR, not only may support diagnosis but also may provide relevant information for prognosis and treatment guidance.

## Introduction

Intramyocardial dissection (ID) is an extremely rare complication of acute myocardial infarction (AMI). Scarce evidence is available in the literature regarding this disorder, mainly limited to isolated case reports.^1–5^ On top of a high level of clinical suspicion, detection of characteristic findings on transthoracic echocardiography (TTE)^1,4^ is essential to establish the diagnosis. However, multimodality imaging may be useful to confirm and to evaluate progression of this complication. This condition may be diagnosed in different clinical stages, ranging from the intramyocardial dissecting haematoma (IMDH) in the early phases to the false-pseudoaneurysm in more severe presentations. Therefore, we report a case series of all the consecutive ID recently managed in our hospital network with a special focus in multimodality imaging (*Summary figure*).

## Summary figure

Whole spectrum of presentation of intramyocardial dissection evaluated with different cardiac imaging diagnostic tools. Column *A*: TTE apical four-chamber (4C) view basal (top) and after contrast (bottom) showing IMDH. Column *B*: cardiac CT 4C views of IMDH at venous (top) and arterial (bottom) phases ruling out perfusion. Column *C*: TTE apical 4C (top) and three-chamber with colour (bottom) views that demonstrate the endocardial flap with flow of an ID. Column *D*: CMR short-axis views with cine (top) and LGE (bottom) views showing the features of an IMDH.

**Figure:**
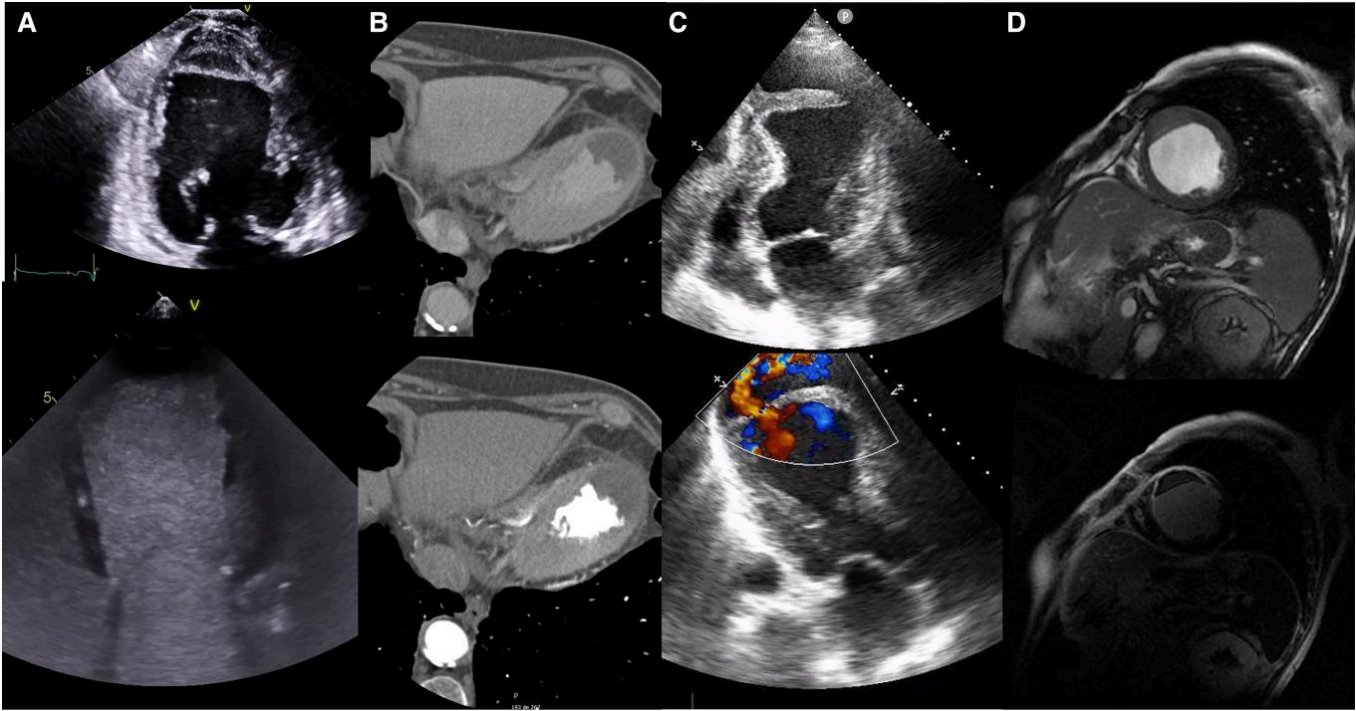


## Patient 1

**Figure 1:**
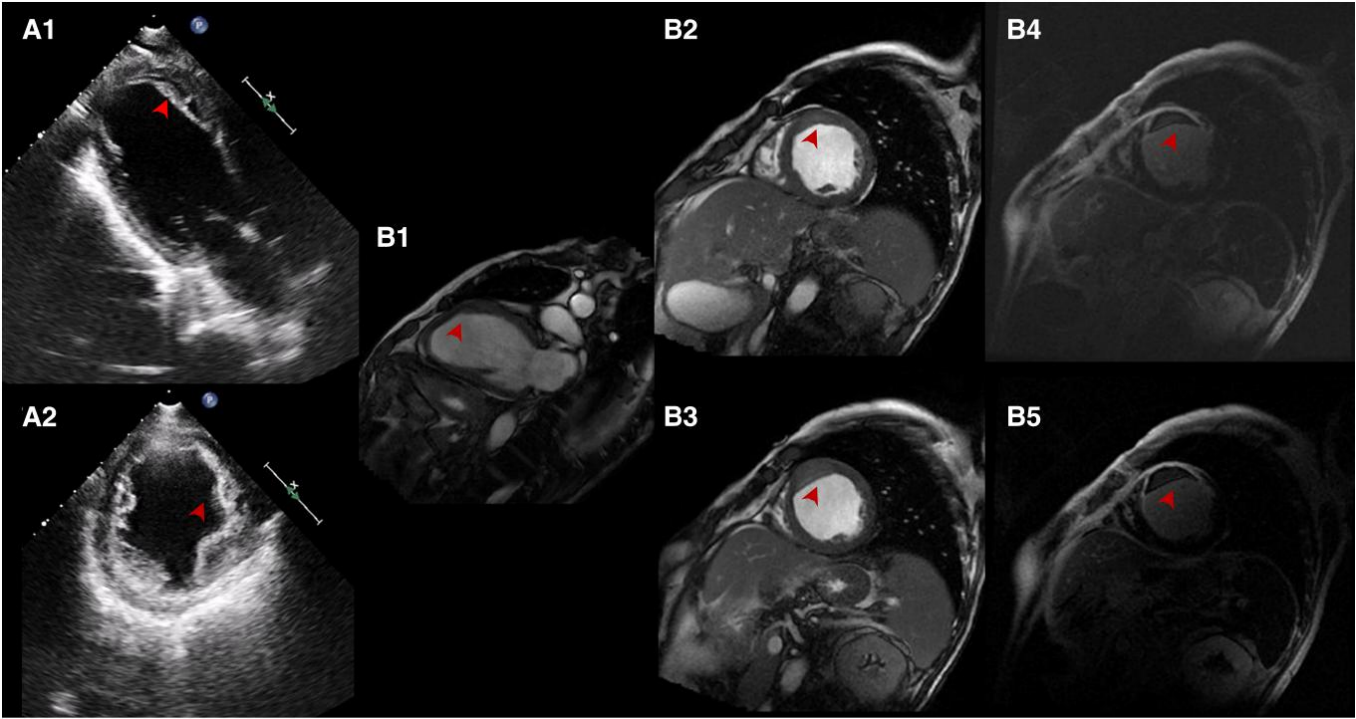
Multimodality cardiac imaging in Patient 1. TTE apical two-chamber (A2C) (*A1*) and short-axis (SA) (*A2*) views show a flap-like endocardial border separation compatible with an IMDH (arrowheads). CMR balanced steady-state free precession (bSSFP) cine A2C (*B1*) and SA (*B2* and *B3*) sequences early after gadolinium contrast administration: note that the apical semilunar shaped IMDH (arrowheads) is separated from the LV, by a ‘flap-like’ structure. LGE SA images (*B4* and *B5*) demonstrate hypointense and surrounded by hyperintense myocardium at the anterior and apical cavity as well as transmural infarction (arrowheads). Note that the aforementioned ‘flap-like’ structure appears as a thin hyper-enhanced layer corresponding to the endocardial border.

Our first patient was a 63-year-old man with history of smoking and sporadic cocaine use. He was referred to the Emergency Department (ED) due to a three-week medical history of progressive exertional angina. Electrocardiogram (ECG) showed ST-segment elevation as well as Q waves in V3–V6 leads. An emergent coronary angiogram was performed revealing a proximal left anterior descending (LAD) artery total occlusion. Percutaneous revascularization was discarded at that moment due to the subacute course of the condition and the spontaneous resolution of the angina. In the next hours, the patient developed cardiogenic shock (CS) requiring high-doses of vasoactive drugs, sedation, and orotracheal intubation. Transthoracic echocardiography evidenced severe LV systolic dysfunction (LVSD), with an estimated LV ejection fraction (LVEF) of 15%, secondary to an extensive anteroseptal and apical akinesia. Additionally, a well-defined hypermobile endocardial flap-like separation with no internal flow on colour Doppler, compatible with IMDH, was detected along the anterior wall (*Figure 1A*, Supplementary material online, *Video 1*). A contrast-enhanced cardiac magnetic resonance (CMR) was performed to assess myocardial viability and IMDH evolution one month later. Apart from persistence of severe LVSD (LVEF 15%), post-contrast imaging revealed both anterior and apical transmural hyper-enhancement and a large cavity clearly separated from the LV by a thin ‘flap-like’ structure corresponding to the fibrotic hyper-enhanced endocardium (*Figure 1B*, Supplementary material online, *Videos 2 and 3*). After a 45-day hospitalization, requiring exclusively supportive treatment, the patient could finally be discharged. He has completed physical rehabilitation and is on follow-up by HF unit in NYHA class II.

## Patient 2

**Figure 2:**
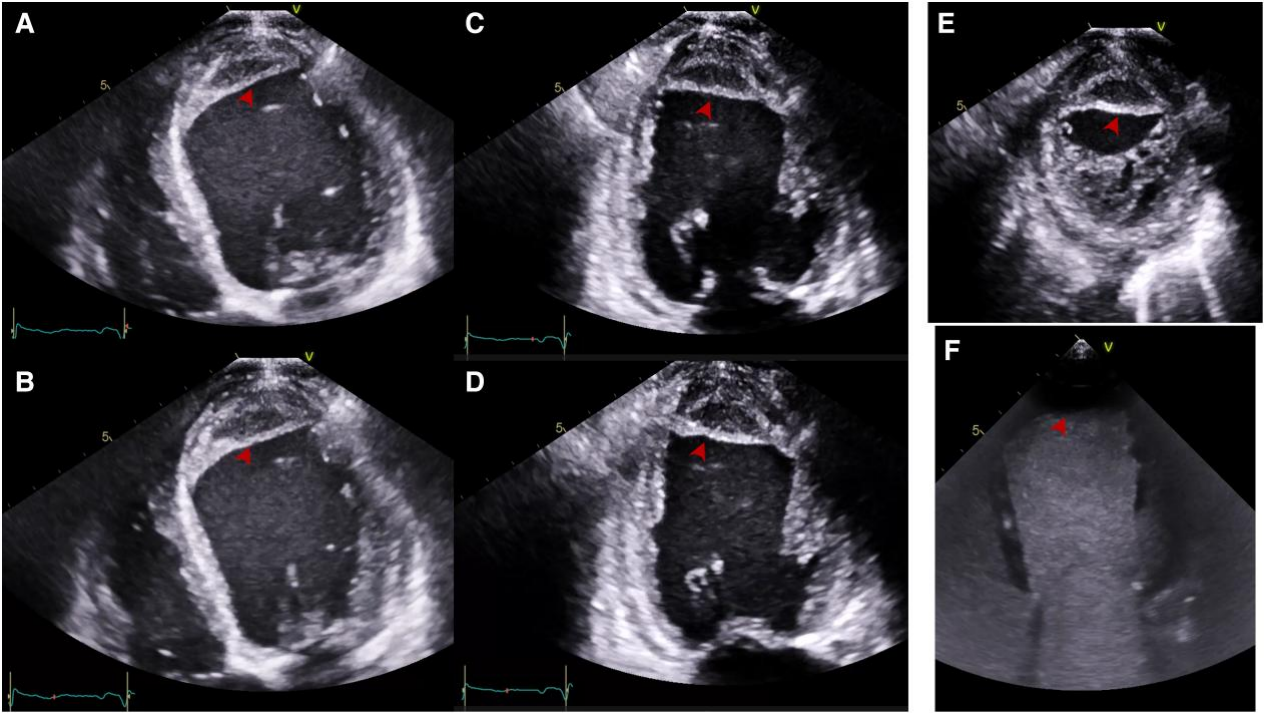
TTE in Patient 2. End-diastole (ED) and end-systole (ES) apical four-chamber (A4C) views (*A* and *B*, respectively). ED (*C*) and ES (*D*) A2C views. Note the systolic expansion of the intramyocardial neo-cavity, compatible with a large anteroapical IMDH (arrowheads). Apical short-axis (SA) view of flap-like endocardial border (*E*). Contrast TTE in A4C view (*F*) shows lack of contrast flow from the LV.

The second patient was a 92-year-old man with previous AMI 5 years ago at another institution. Although limited information was available about this event, it was known that a three-vessel coronary artery disease (CAD) was observed in coronary angiography and he underwent percutaneous revascularization of the LAD. Moreover, he developed paroxysmal atrial fibrillation in the acute setting but he was discharged on dual antiplatelet therapy only. Follow-up TTE reported an LVEF of 40% with no other relevant findings. At the index hospitalization, he was admitted due to heart failure (HF), renal failure, and acute confusional syndrome compatible with a CS status. The anamnesis was unspecific but EKG changes and cardiac biomarkers elevation were compatible with evolved myocardial infarction. A new TTE showed a severe LV systolic dysfunction due to wall motion abnormalities and a large anteroapical IMDH (*Figure 2*, Supplementary material online, *Video 4*). In addition, severe mixed aortic valve disease as well as severe mitral and tricuspid regurgitation was noted. Given the late diagnosis, the advanced age of the patient and the refractoriness of the CS, with deterioration despite vasopressor treatment, conservative management was considered and comfort measures prevailed. The patient died in the following days.

## Patient 3

**Figure 3:**
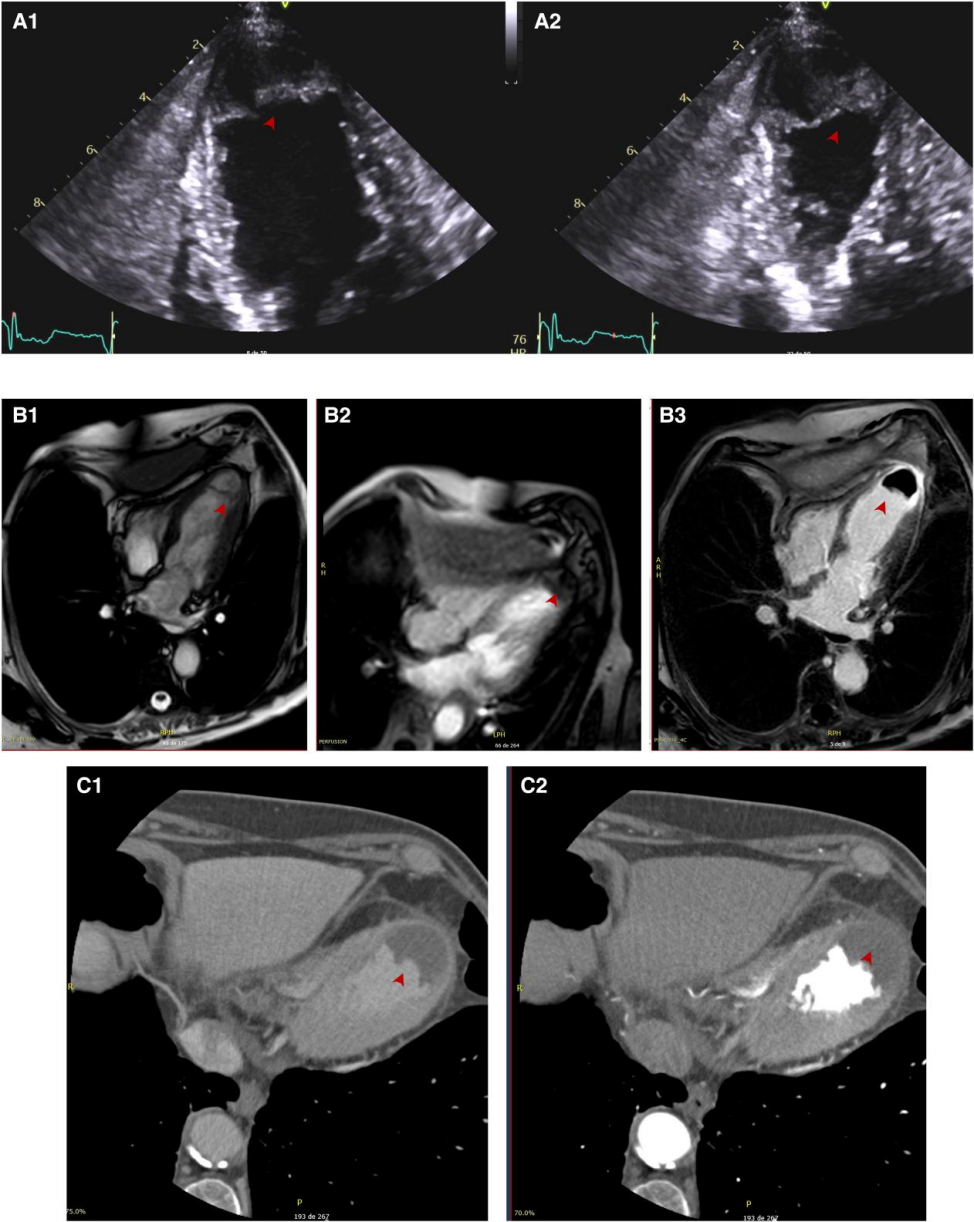
Multimodality imaging in Patient 3. TTE ED and ES A4C views (*A1* and *A2*) show an apical hypoechoic mass, initially considered to be an intraventricular thrombus (arrowheads). CMR bSSFP cine sequences before (*B1*) and early after (*B2*) gadolinium contrast administration: note that the apical cavity is clearly separated from the LV, by a ‘flap-like’ structure (arrowheads). Such findings rule-out an intraventricular thrombus. LGE images (*B3*) demonstrate hypointense and surrounded by hyperintense myocardium at the apical cavity as well as absence of viability (arrowheads). Pre-contrast (*C1*) and contrast-enhanced (*C2*) cardiac CT images: apical repletion defect without any contrast flow from the LV cavity (arrowheads).

Our third patient was an 82-year-old woman with hypertension and dyslipidaemia as cardiovascular risk factors, and previous history of non-Hodgkin’s lymphoma and severe chronic obstructive pulmonary disease. She was referred to de ED due to recent onset intermittent atypical chest pain, and the ECG showed complete right bundle branch block and ST-segment elevation in precordial leads. No relevant findings were found in physical examination. Transthoracic echocardiography demonstrated severe LVSD (LVEF of 34%) with significant myocardial thinning and akinesia of the apical, mid-anterior and mid-septal segments. A suspicious image was observed in the apical area, which was initially considered to be an intraventricular thrombus (*Figure 3A*). Therefore, an extensive evolved anteroseptal AMI was diagnosed and coronary angiogram was performed showing a mid-LAD total occlusion. As the next diagnostic step, CMR was performed, which confirmed severe LV dysfunction and showed a myocardial wall tear within the apex conforming a 3 cm thickness cavity. This region presented hyperintensity in the T2-weighted sequences, consistent with myocardial oedema. Late gadolinium enhancement (LGE) demonstrated a hypointense area surrounded by hyperintense myocardium at the apical cavity as well as absence of viability in the aforementioned akinetic segments (*Figure 3B*). Although these findings were already diagnostic for ID, a contrast-enhanced cardiac computed tomography (CT) was requested for a further evaluation. This technique identified an apical repletion defect in proximity to the myocardium without any contrast flow from the LV cavity (*Figure 3C*). Following this multimodality imaging approach, the diagnosis of thrombosed IMDH was confirmed. The patient was stable from the clinical point of view and was considered non-candidate for intervention due to high surgical risk and absence of myocardial viability. Antiplatelet therapy was discontinued, and the patient was discharged on medical treatment in good functional condition. During the last years, she has been followed by the HF unit without any noticeable cardiovascular decompensation.

## Patient 4

**Figure 4:**
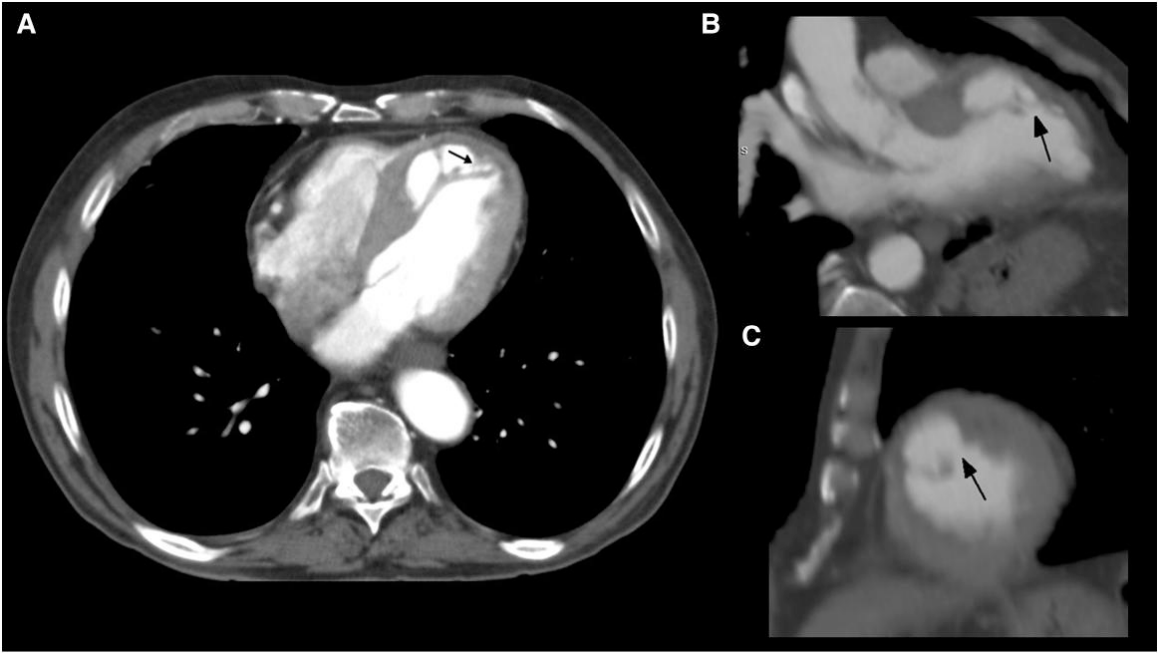
Cardiac CT images in Patient 5. A4C (*A*), A3C (*B*), and SA (*C*) views after multiplanar reconstruction. We note an intramyocardial dissecting cavity communicating with the LV through a narrow neck (arrows).

The fourth patient was a 72-year-old man with previous anteroseptal AMI 16 years ago complicated with an IMDH who was asymptomatic at the time of consultation. As part of a routine follow-up and given the fact that no advanced imaging scans had been performed ever since, a cardiac CT was requested. The multiplanar views showed an anteroseptal intramyocardial cavity communicating with the LV through a narrow neck, consistent with a chronic false-pseudoaneurysm without any progression in terms of size and shape (*Figure 4*). Despite advanced imaging evaluation, conservative medical treatment was continued without changes.

## Patient 5

**Figure 5:**
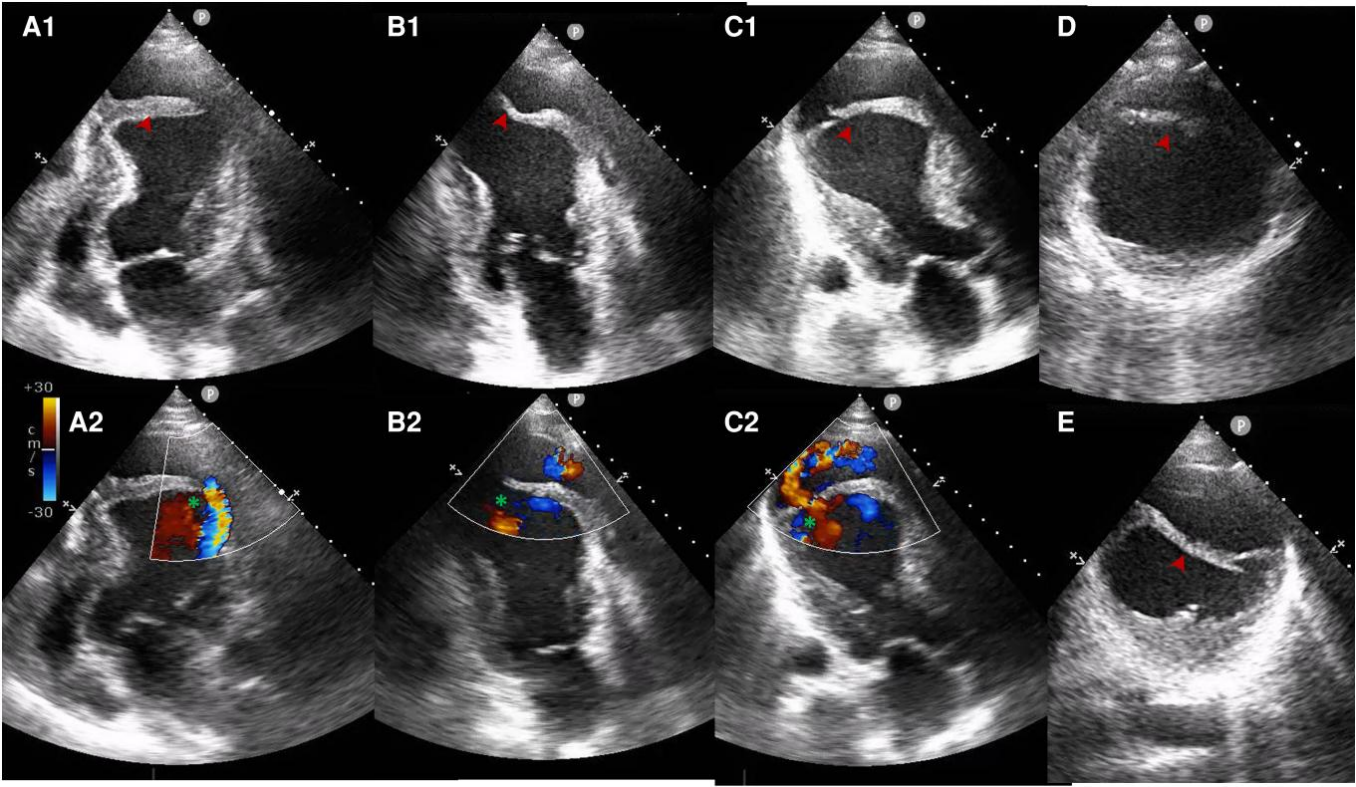
TTE in Patient 4. A4C (*A1*), A2C (*B1*), and apical three-chamber (A3C) views (*C1*) show a very large apical, anterior, and anteroseptal ID. We note a ‘flap-like’ structure separating the neo-cavity from the LV with a clear solution of continuity (arrowheads). Doppler images in the aforementioned views (*A2*, *B2*, and *C2*, respectively) show bidirectional flow (asterisk) between the LV and the IMDH. This continuous high pressure blood flow is with high level of suspicion the cause for the rapid and outstanding growth of the false-pseudoaneurysm.

**Figure 6:**
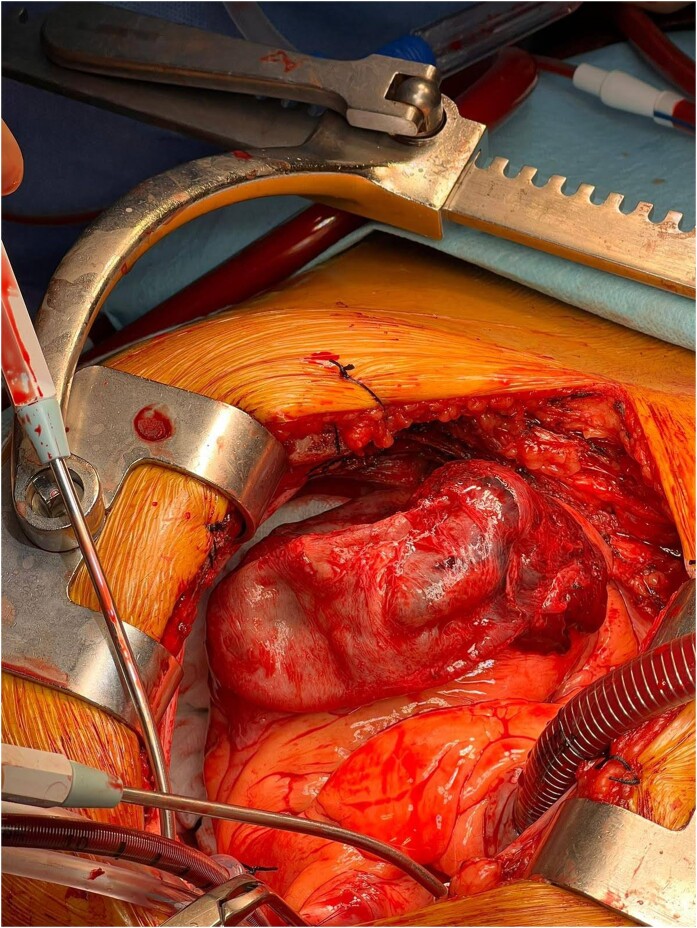
Intraoperative view after thoracotomy. The continuity of the epicardial wall can be appreciated without intrapericardial haematoma. This fact rules out a pseudoaneurysm and confirms the diagnosis of an ID open to the ventricular chamber (false-pseudoaneurysm).

Our fifth patient was a 47-year-old man with medical history of hypertension, dyslipidaemia, type II diabetes, and sporadic cannabis consumption. He was referred to the ED due to syncope at rest and new-onset chest pain and dyspnoea. Clinically, he presented with severe HF, respiratory failure, and pre-CS signs such as tachycardia, hypotension, and increased blood lactate levels. Electrocardiogram showed anterior ST-segment elevation and established Q waves. Transthoracic echocardiography reported a severe LVSD (LVEF 30%) due to apical dyskinesia and akinesia of the mid-septal segments, a mild pericardial effusion without tamponade, and a large apical cavity considered to be a false-pseudoaneurysm (*Figure 5*, Supplementary material online, *Video 5*). Initial medical treatment with non-invasive positive pressure ventilation, intravenous furosemide, and vasoactive drugs was started, and an emergent surgical treatment was indicated. A *Dor* procedure was performed, consisting in a LV reconstruction with an endoventricular circular pericardial patch plasty.^6^ The surgeon reported a severely calcified LAD and a highly friable myocardial tissue (*Figure 6*). After an 11-day hospitalization, the patient could be discharged and he is on follow-up by HF unit in NYHA class I for more than 2 years.

## Discussion

Intramyocardial dissection is a very rare MI mechanical complication caused by endothelial dysfunction of the intramyocardial vessel, which leads to increased capillary permeability, elevated intraventricular pressure, and decreased tissue tension forces in the ischaemic region.^1,5^ These conditions conduct to the development of cavitated tracts and lacunae within the myocardial wall filled with blood following an irregular and anfractuous path. These tracts are externally limited by the pericardium and part of the myocardial wall and internally by the endocardium and the rest of the myocardial fibre layers. Therefore, it would constitute an uncommon type of subacute incomplete and contained myocardial rupture.

As illustrated by our five cases, it might be diagnosed in different clinical and evolutive presentations. In the early stages, it would present as an IMDH also described as ‘closed ID’. The defining characteristic of this stage is the absence of communication with the LV chamber. In more severe or advanced phases, the ID tears into the LV cavity and a false-pseudoaneurysm or ‘open dissection’ would be found. This entity can be found in the literature as ‘pseudo-falseaneurysm’ or ‘pseudopseudoaneurysm’, a nomenclature that may be confusing.^7,8^ We propose ‘open intramyocardial dissection’ as a unique and more comprehensive term. Moreover, differential diagnosis with pseudoaneurysm might be challenging and can only be made if the continuity of the myocardial wall surrounding the dissecting chamber is proven.

Typical locations of ID are LV free wall, interventricular septum (IVS), and right ventricle.^4^ The most common presentation is the one involving the LV free wall,^5^ as observed in all of our patients. Eventually, it may be further complicated by rupture of the LV free wall or the IVS. Mortality rate varies between 33% and 47% depending on published series.^1,5^ Better survival has been reported in cases involving the LV free wall,^5^ while mortality can reach 78% when the IVS is affected.^4^ Both an LVEF under 35% and an age over 60 years have been suggested as independent predictors of in-hospital death.^1^

On echocardiography, this entity appears as one or multiple intramyocardial pulsatile neo-cavities with systolic expansion. Such cavities are delimited by a thin and hypermobile endocardial border that simulates a ‘flap’ and show hypoechoic heterogeneous content.^1^ The presence of a higher echogenicity heterogeneous content is suggestive of partially or totally thrombosed haematic component.^4^ Echocardiographic contrast may assist in the diagnosis of this entity. Unlike in other mechanical complications, it will demonstrate a continuity in the endocardial border in closed ID or entrance of contrast within a myocardial cavity in open ID.

Differential diagnosis should include ventricular pseudoaneurysm and intraventricular thrombus, as seen in Patient 3. While traditionally diagnosis was made by TTE and transoesophageal echocardiography,^4^ nowadays, advanced cardiac imaging techniques play a fundamental role.^2,5^ Therefore, CMR is the gold standard for the diagnosis of this condition, showing the typical flap-like endocardial tear in cine sequences and hypointense area surrounded by hyperintense myocardium in LGE (Patients 1 and 3).^1^ In contrast, ventricular pseudoaneurysm appears as a myocardial wall solution of continuity resulting in a cavity with a neck-to-depth ratio < 50%, more frequently affecting the basal inferior or inferolateral wall. Whereas intraventricular thrombus is seen as an irregular-shaped endoluminal mass with no surrounding endocardial border or perfusion, which shows a post-contrast pattern of hyperintensity/isointensity with short inversion time (IT) and hypointensity with long IT in Look-Locker sequence.^9^

The best management for this AMI complication is not clearly established, and the medical vs. surgical approach remains controversial. The current acute coronary syndromes guidelines do not include any reference nor indication to this potential complication.^10^ Thus, it will be necessary to individualize the treatment of each particular case.^3^ In our series, the first four patients were managed medically due to haemodynamic stability, absence of pericardial effusion or IVS defects, but also due to poor prognosis (Patient 2). It has been reported that IMDH limited to the apex trends to spontaneously reabsorb in up to 30% of cases (Patient 3),^4,5^ so an initial conservative approach is reasonable. On the other hand, if a large false-pseudoaneurysm is present, the RV free wall is involved, or if a rupture of the LV free wall or IVS is present, urgent cardiac surgery is indicated. Consequently, in the fifth patient, urgent surgical treatment was performed due to a very high risk of cardiac rupture secondary to the large extension of the ID and the evidence of pericardial effusion.

## Conclusion

Intramyocardial dissection is a rare entity that should be considered among the mechanical complications of AMI. Detection of typical echocardiographic findings is paramount but, as highlighted in our series, multimodal imaging may be necessary for differential diagnosis and provide relevant information for individualized therapeutic management.

## Supplementary Material

ytae219_Supplementary_Data

## Data Availability

In order to keep confidentiality, original medical records and complementary test are not provided along with this work.

## Lead author biography

Irene Carrión-Sánchez is a medical doctor graduate from the University Complutense (Madrid, Spain). After completing the Cardiology Residency Program at the Clínico San Carlos Hospital, she has recently joined a Cardiovascular Imaging Fellowship at the Ramón y Cajal Hospital (Madrid, Spain). Her main fields of interest are advanced cardiac imaging and valvular heart disease.

## Supplementary material

**Consent:** Informed consent was obtained from the patients included in the present article following COPE and ICMJE recommendations.

**Funding:** None declared.
