# Supplementary material for: Role of multimodality cardiac imaging for evaluation of intramyocardial dissection, from dissecting haematoma to false-pseudoaneurysm: a case series
Source: Eur Heart J Case Rep. 2024 May 7;8(5):ytae219. doi: 10.1093/ehjcr/ytae219 (PMC11091479; doi:10.1093/ehjcr/ytae219)
Supplement: ytae219_Supplementary_Data [file ytae219_supplementary_data.zip › Supplementary Video captions.docx]

VIDEOS

Video 1. Transthoracic echocardiogram in 2-chambers view from patient #1. There is a flap-like endocardial border separation compatible with an IMDH in the mid anterior wall.

Video 2. Cardiac magnetic cine sequences in 2-chambers projection from patient #1. A non-contractile thickening of the mid anterior LV segment with hypointensity is noted corresponding to IMDH.

Video 3. Cardiac magnetic cine sequences in short axis projection at mid-to-apical level from patient #1. Ortogonal view of the aforementioned finding.

Video 4. Contrast transthoracic echocardiogram in 4-chambers view from patient#2. There is a filling defect along the apex showing continuity with endocardial border, which suggests IMDH.

Video 5. Transthoracic echocardiogram in 3-chambers view with colour from patient#5. Please note the presence of an endocardial flap in the left ventricular apex with an entry that shows bidirectional flow.
